# Supplementary material for: Niche-specific genome degradation and convergent evolution shaping Staphylococcus aureus adaptation during severe infections
Source: eLife. 2022 Jun 14;11:e77195. doi: 10.7554/eLife.77195 (PMC9270034; doi:10.7554/eLife.77195)
Supplement: Table 1—source data 1. [file elife-77195-table1-data1.zip › Table1source_data1.docx]

## Table 1 – source data 1. List of within-host studies included in the analysis.

| Author, year | PMID | N independent  episodes | N sequences | Ref |
| --- | --- | --- | --- | --- |
| Gao W, 2015 | 28348811 | 1 | 18 | (1) |
| Howden BP, 2011 | 22102812 | 5 | 10 | (2) |
| Young BC, 2012 | 22393007 | 3 | 167 | (3) |
| Golubchik, 2013 | 23658690 | 13 | 120 | (4) |
| Burd EM, 2014 | 24850355 | 1 | 3 | (5) |
| Rishishwar L, 2016 | 27446992 | 1 | 3 | (6) |
| Trouillet-Assant S, 2016 | 26918656 | 3 | 6 | (7) |
| Young BC, 2017 | 29256859 | 105 | 1078 | (8) |
| Rouard C, 2018 | 30275089 | 1 | 3 | (9) |
| Langhanki L, 2018 | 30348081 | 1 | 2 | (10) |
| Altman DR, 2018 | 30061376 | 10 | 23 | (11) |
| Giulieri SG, 2018 | 30103826 | 49 | 111 | (12) |
| Benoit JB, 2018 | 29723202 | 8 | 42 | (13) |
| Suligoy CM, 2018 | 29456969 | 1 | 3 | (14) |
| Harkins CP, 2018 | 28951239 | 27 | 248 | (15) |
| Tan X, 2019 | 30753350 | 4 | 8 | (16) |
| Loss G, 2019 | 31696062 | 1 | 2 | (17) |
| Kuroda M, 2019 | 31474962 | 1 | 16 | (18) |
| Azarian T, 2019 | 31244886 | 3 | 44 | (19) |
| Wuethrich D, 2019 | 30726929 | 1 | 5 | (20) |
| Ji S, 2020 | 32176794 | 3 | 20 | (21) |
| Miller CR, 2020 | 31932377 | 1 | 2 | (22) |
| Liu J, 2020 | 31919223 | 2 | 12 | (23) |
| Petrovic Fabijan A, 2020 | 32066959 | 12 | 57 | (24) |
| Tong SYC, 2020 | 32044943 | 138 | 585 | (25) |

## References

1. Gao W, Monk IR, Tobias NJ, Gladman SL, Seemann T, Stinear TP, et al. Large tandem chromosome expansions facilitate niche adaptation during persistent infection with drug-resistant Staphylococcus aureus. Microb Genom. 2015;1(2):e000026.

2. Howden BP, McEvoy CR, Allen DL, Chua K, Gao W, Harrison PF, et al. Evolution of multidrug resistance during Staphylococcus aureus infection involves mutation of the essential two component regulator WalKR. PLoS Pathog. 2011;7(11):e1002359.

3. Young BC, Golubchik T, Batty EM, Fung R, Larner-Svensson H, Votintseva AA, et al. Evolutionary dynamics of Staphylococcus aureus during progression from carriage to disease. Proc Natl Acad Sci U S A. 2012;109(12):4550-5.

4. Golubchik T, Batty EM, Miller RR, Farr H, Young BC, Larner-Svensson H, et al. Within-host evolution of Staphylococcus aureus during asymptomatic carriage. PLoS One. 2013;8(5):e61319.

5. Burd EM, Alam MT, Passalacqua KD, Kalokhe AS, Eaton ME, Satola SW, et al. Development of oxacillin resistance in a patient with recurrent Staphylococcus aureus bacteremia. J Clin Microbiol. 2014;52(8):3114-7.

6. Rishishwar L, Kraft CS, Jordan IK. Population Genomics of Reduced Vancomycin Susceptibility in Staphylococcus aureus. mSphere. 2016;1(4).

7. Trouillet-Assant S, Lelievre L, Martins-Simoes P, Gonzaga L, Tasse J, Valour F, et al. Adaptive processes of Staphylococcus aureus isolates during the progression from acute to chronic bone and joint infections in patients. Cell Microbiol. 2016;18(10):1405-14.

8. Young BC, Wu C-H, Gordon NC, Cole K, Price JR, Liu E, et al. Severe infections emerge from commensal bacteria by adaptive evolution. eLife. 2017;6:e30637.

9. Rouard C, Garnier F, Leraut J, Lepainteur M, Rahajamananav L, Languepin J, et al. Emergence and Within-Host Genetic Evolution of Methicillin-Resistant Staphylococcus aureus Resistant to Linezolid in a Cystic Fibrosis Patient. Antimicrob Agents Chemother. 2018;62(12).

10. Langhanki L, Berger P, Treffon J, Catania F, Kahl BC, Mellmann A. In vivo competition and horizontal gene transfer among distinct Staphylococcus aureus lineages as major drivers for adaptational changes during long-term persistence in humans. BMC Microbiol. 2018;18(1):152.

11. Altman DR, Sullivan MJ, Chacko KI, Balasubramanian D, Pak TR, Sause WE, et al. Genome Plasticity of agr-Defective Staphylococcus aureus during Clinical Infection. Infect Immun. 2018;86(10).

12. Giulieri SG, Baines SL, Guerillot R, Seemann T, Gonçalves da Silva A, Schultz M, et al. Genomic exploration of sequential clinical isolates reveals a distinctive molecular signature of persistent Staphylococcus aureus bacteraemia. Genome Medicine. 2018;10(1):65.

13. Benoit JB, Frank DN, Bessesen MT. Genomic evolution of Staphylococcus aureus isolates colonizing the nares and progressing to bacteremia. PLoS One. 2018;13(5):e0195860.

14. Suligoy CM, Lattar SM, Noto Llana M, Gonzalez CD, Alvarez LP, Robinson DA, et al. Mutation of Agr Is Associated with the Adaptation of Staphylococcus aureus to the Host during Chronic Osteomyelitis. Front Cell Infect Microbiol. 2018;8:18.

15. Harkins CP, Pettigrew KA, Oravcova K, Gardner J, Hearn RMR, Rice D, et al. The Microevolution and Epidemiology of Staphylococcus aureus Colonization during Atopic Eczema Disease Flare. J Invest Dermatol. 2018;138(2):336-43.

16. Tan X, Coureuil M, Ramond E, Euphrasie D, Dupuis M, Tros F, et al. Chronic Staphylococcus aureus Lung Infection Correlates With Proteogenomic and Metabolic Adaptations Leading to an Increased Intracellular Persistence. Clin Infect Dis. 2019;69(11):1937-45.

17. Loss G, Simoes PM, Valour F, Cortes MF, Gonzaga L, Bergot M, et al. Staphylococcus aureus Small Colony Variants (SCVs): News From a Chronic Prosthetic Joint Infection. Front Cell Infect Microbiol. 2019;9:363.

18. Kuroda M, Sekizuka T, Matsui H, Ohsuga J, Ohshima T, Hanaki H. IS256-Mediated Overexpression of the WalKR Two-Component System Regulon Contributes to Reduced Vancomycin Susceptibility in a Staphylococcus aureus Clinical Isolate. Front Microbiol. 2019;10:1882.

19. Azarian T, Ridgway JP, Yin Z, David MZ. Long-Term Intrahost Evolution of Methicillin Resistant Staphylococcus aureus Among Cystic Fibrosis Patients With Respiratory Carriage. Front Genet. 2019;10:546.

20. Wuthrich D, Cuenod A, Hinic V, Morgenstern M, Khanna N, Egli A, et al. Genomic characterization of inpatient evolution of MRSA resistant to daptomycin, vancomycin and ceftaroline. J Antimicrob Chemother. 2019;74(5):1452-4.

21. Ji S, Jiang S, Wei X, Sun L, Wang H, Zhao F, et al. In-Host Evolution of Daptomycin Resistance and Heteroresistance in Methicillin-Resistant Staphylococcus aureus Strains From Three Endocarditis Patients. J Infect Dis. 2020;221(Suppl 2):S243-S52.

22. Miller CR, Dey S, Smolenski PD, Kulkarni PS, Monk JM, Szubin R, et al. Distinct Subpopulations of Intravalvular Methicillin-Resistant Staphylococcus aureus with Variable Susceptibility to Daptomycin in Tricuspid Valve Endocarditis. Antimicrob Agents Chemother. 2020;64(3).

23. Liu J, Gefen O, Ronin I, Bar-Meir M, Balaban NQ. Effect of tolerance on the evolution of antibiotic resistance under drug combinations. Science. 2020;367(6474):200-4.

24. Petrovic Fabijan A, Lin RCY, Ho J, Maddocks S, Ben Zakour NL, Iredell JR, et al. Safety of bacteriophage therapy in severe Staphylococcus aureus infection. Nat Microbiol. 2020;5(3):465-72.

25. Tong SYC, Lye DC, Yahav D, Sud A, Robinson JO, Nelson J, et al. Effect of Vancomycin or Daptomycin With vs Without an Antistaphylococcal β-Lactam on Mortality, Bacteremia, Relapse, or Treatment Failure in Patients With MRSA Bacteremia: A Randomized Clinical Trial. JAMA. 2020;323(6):527-37.
